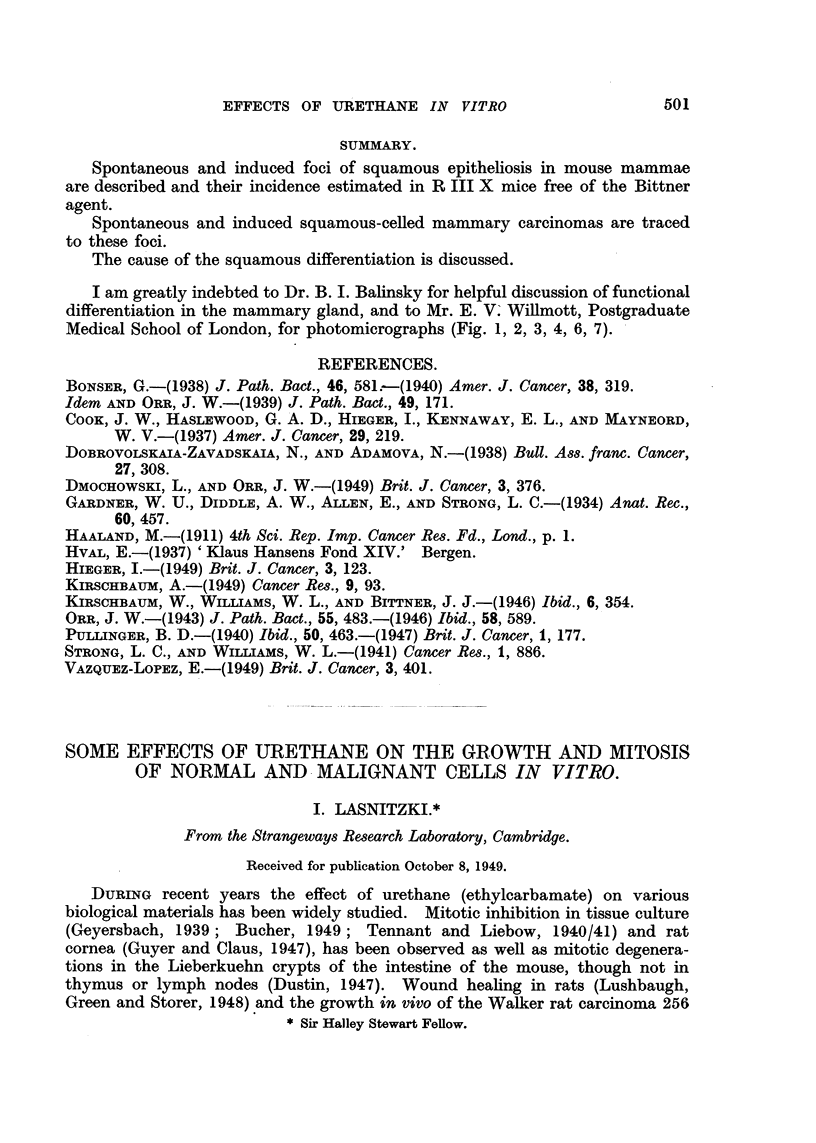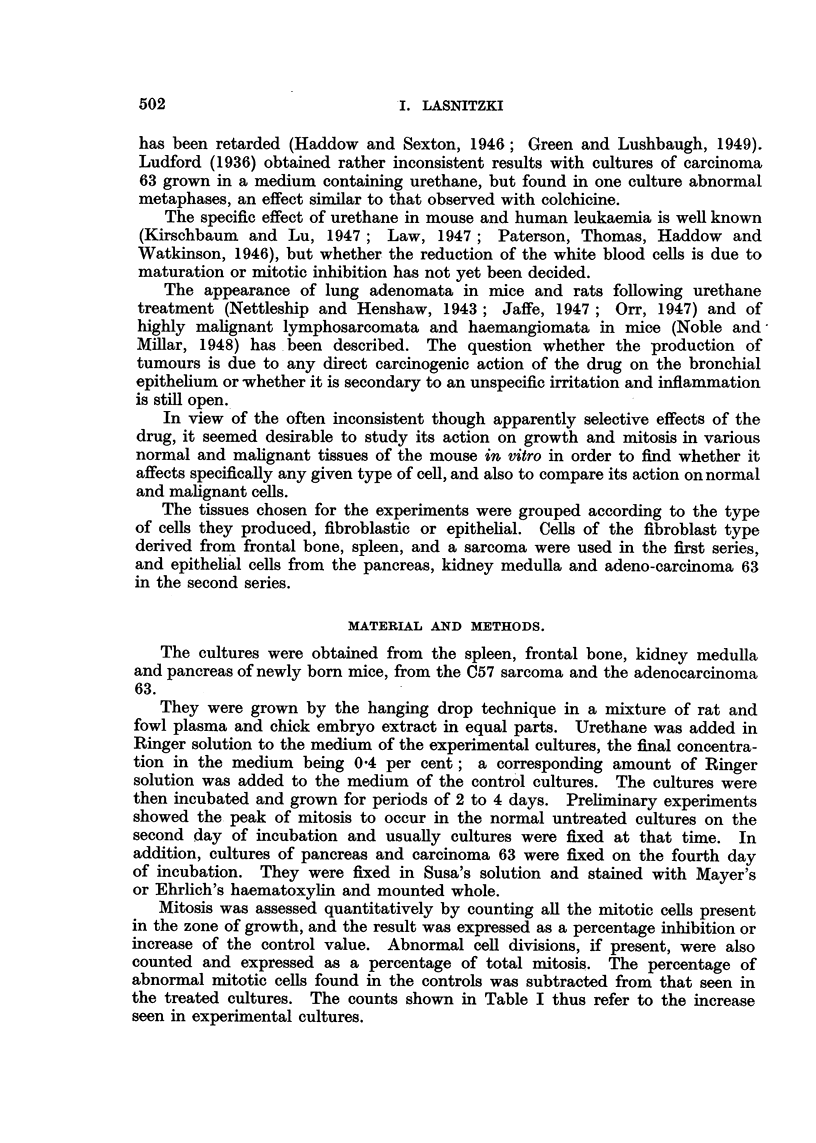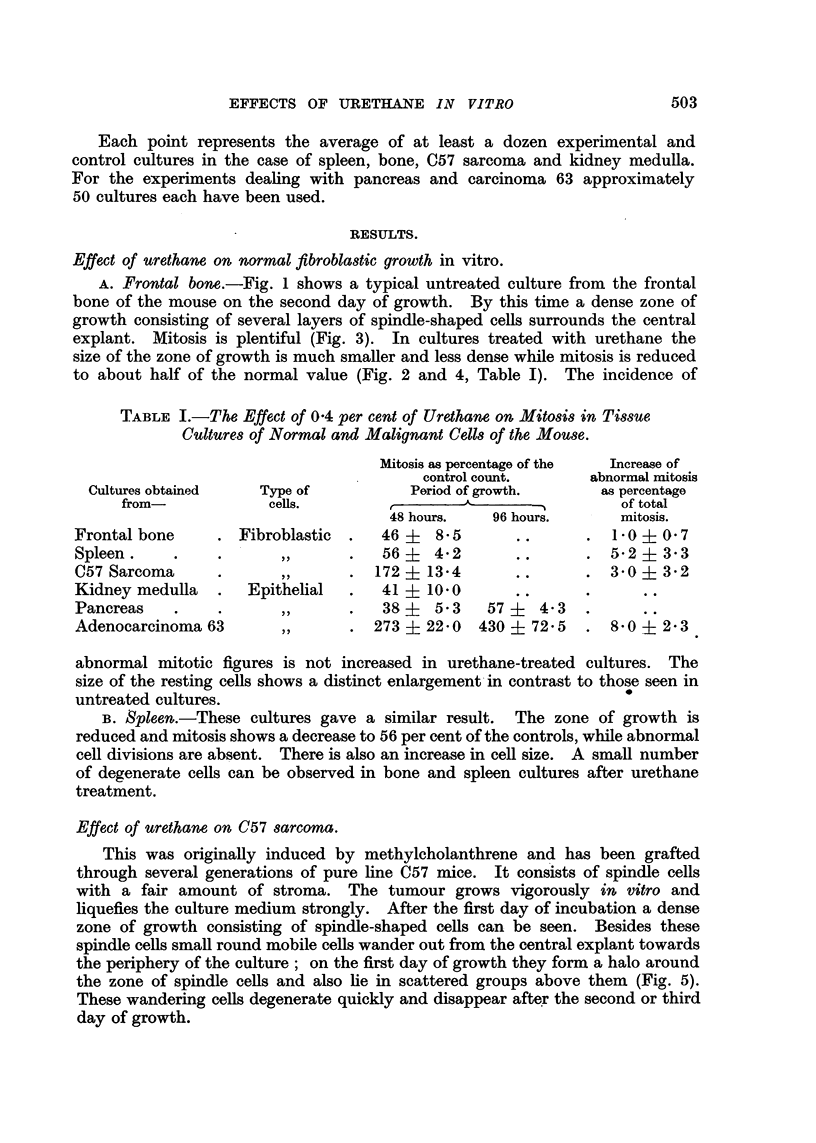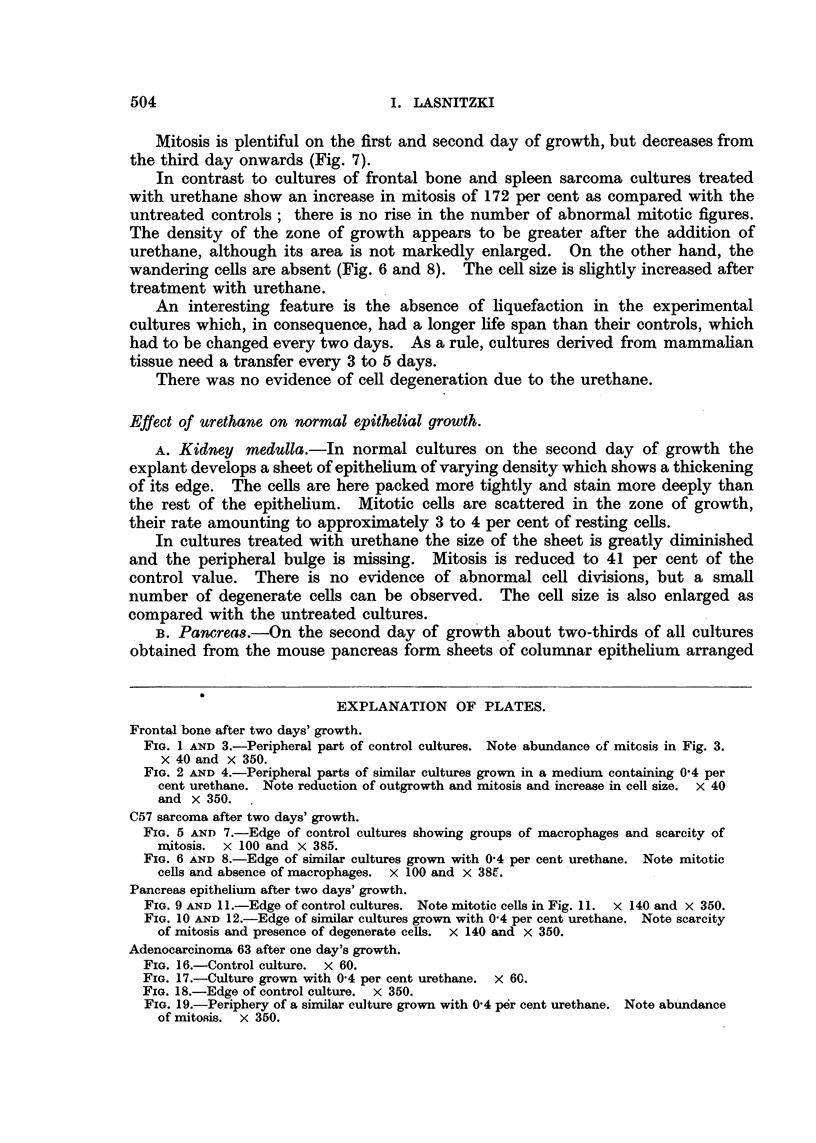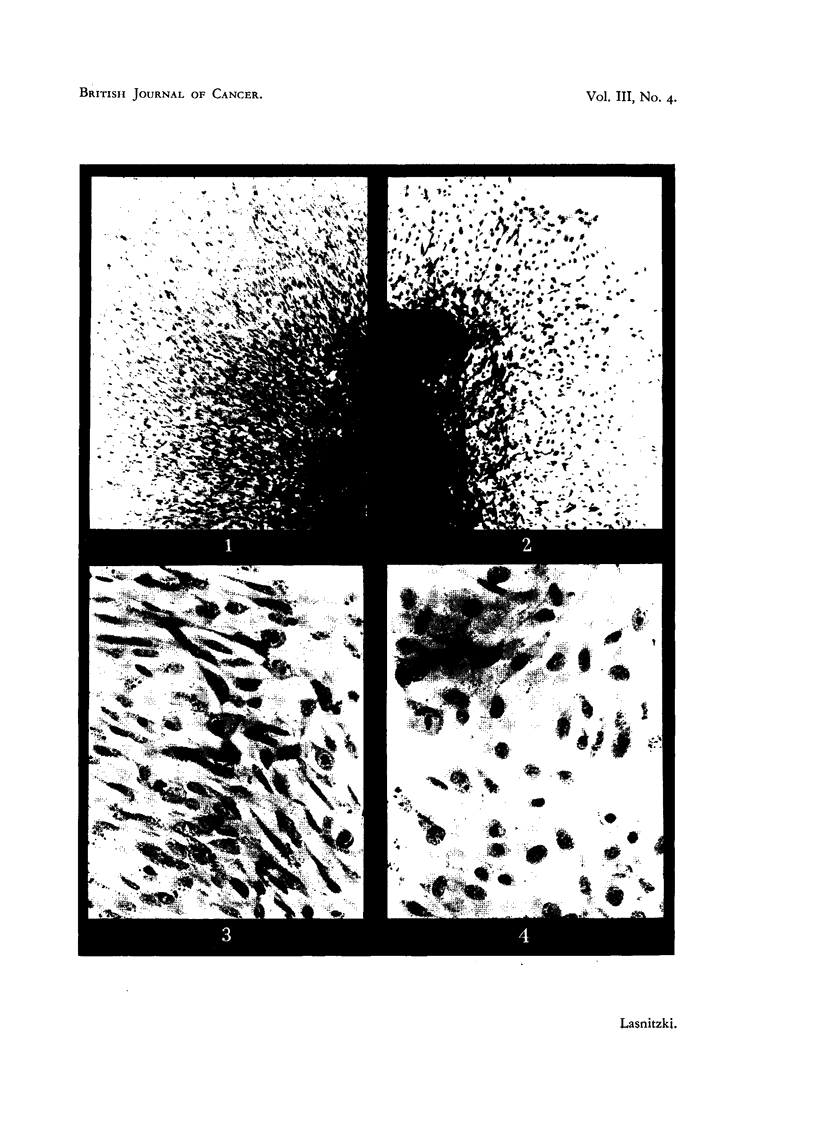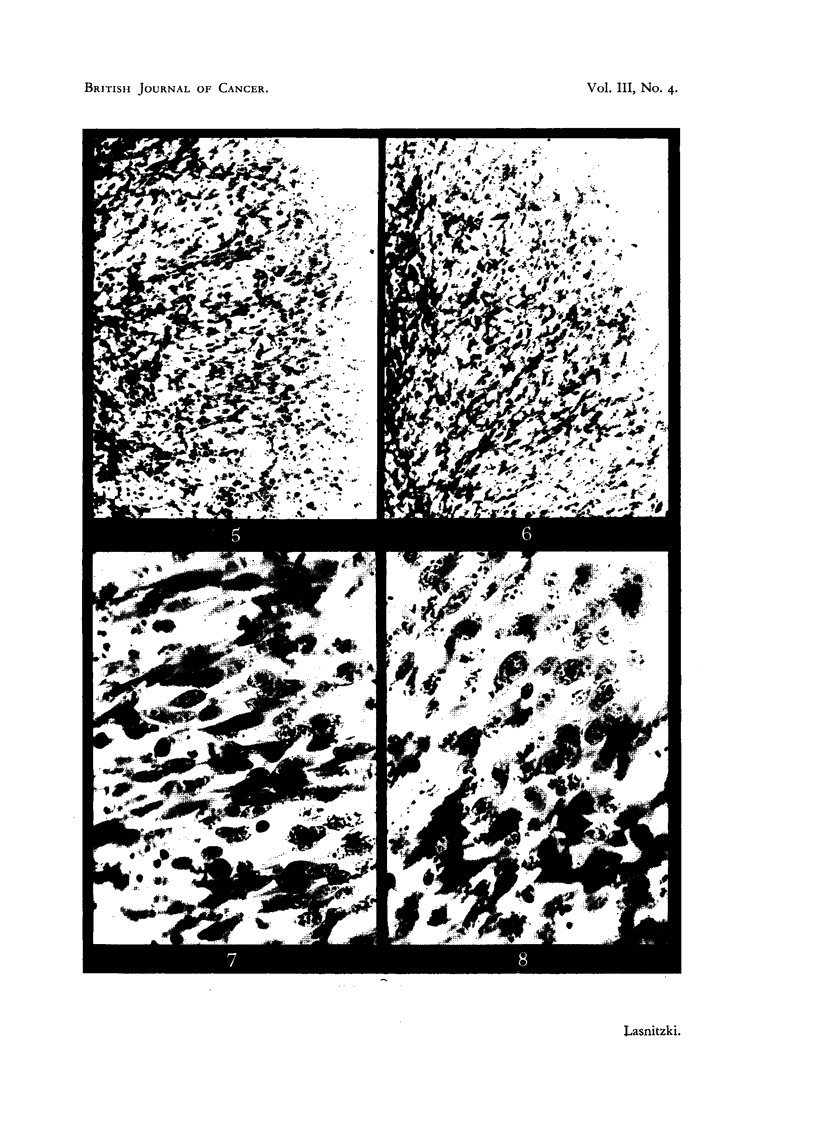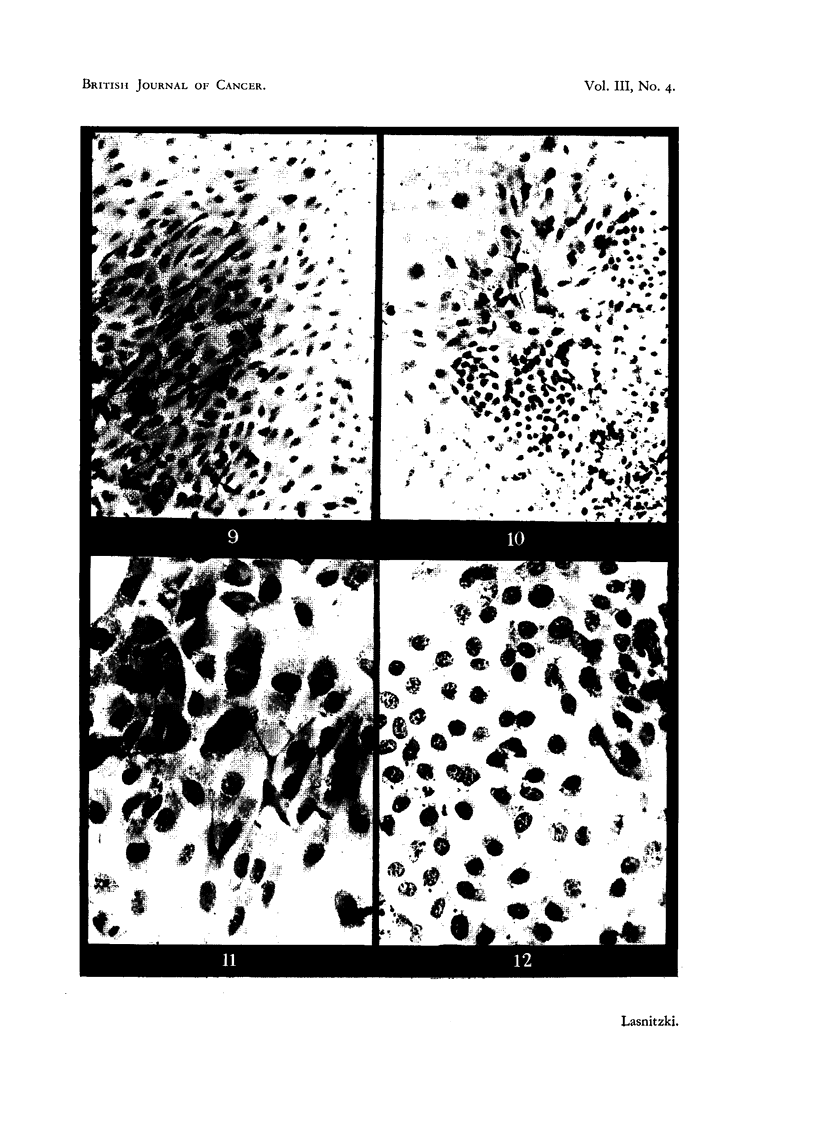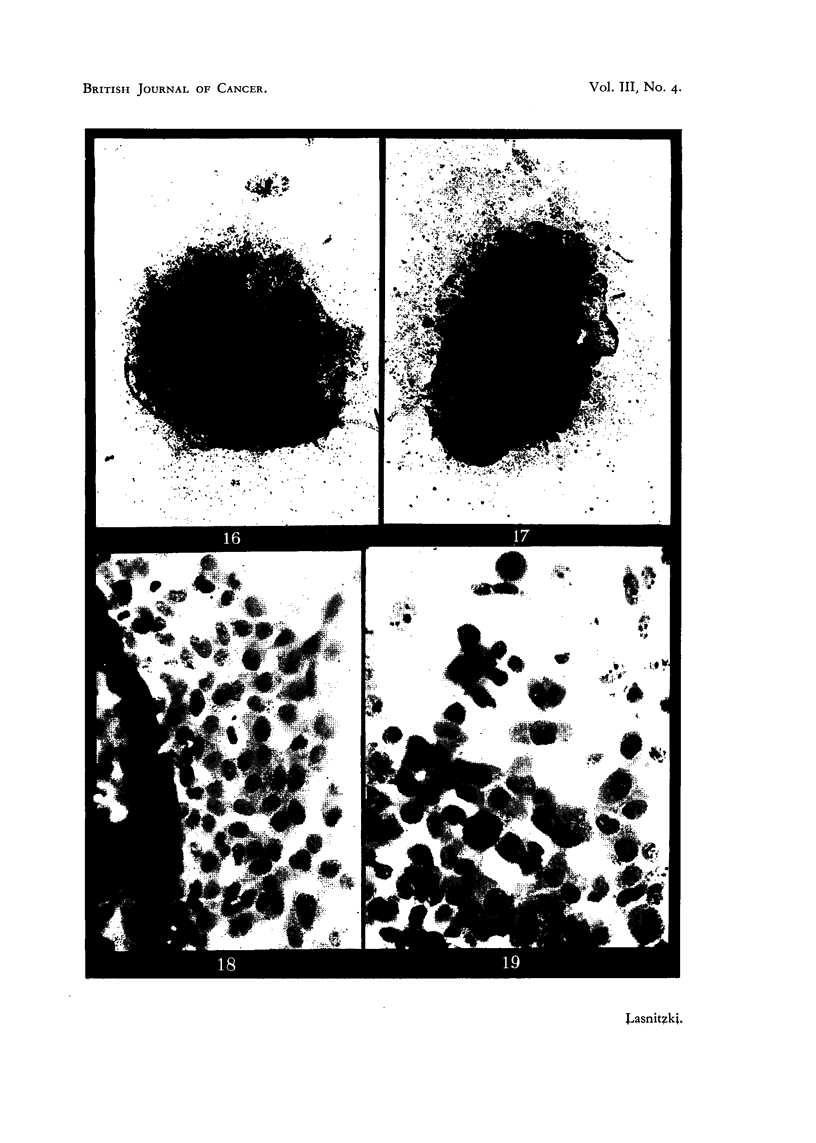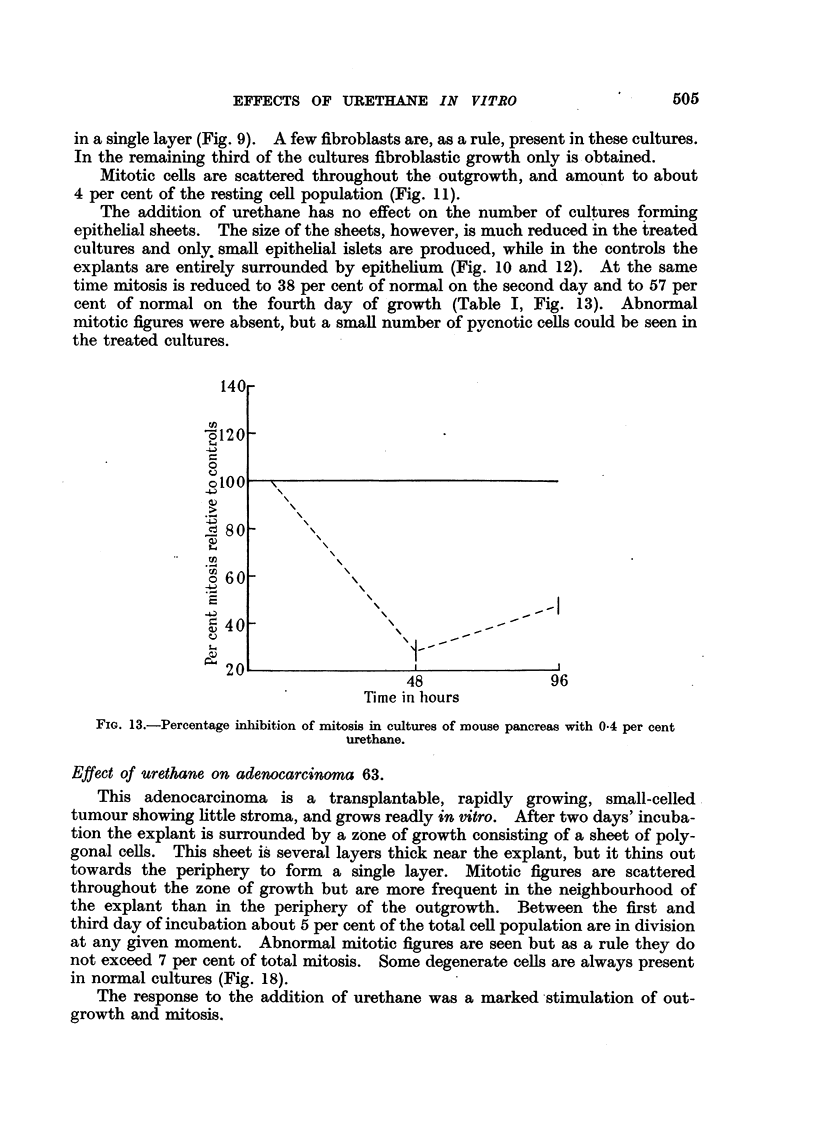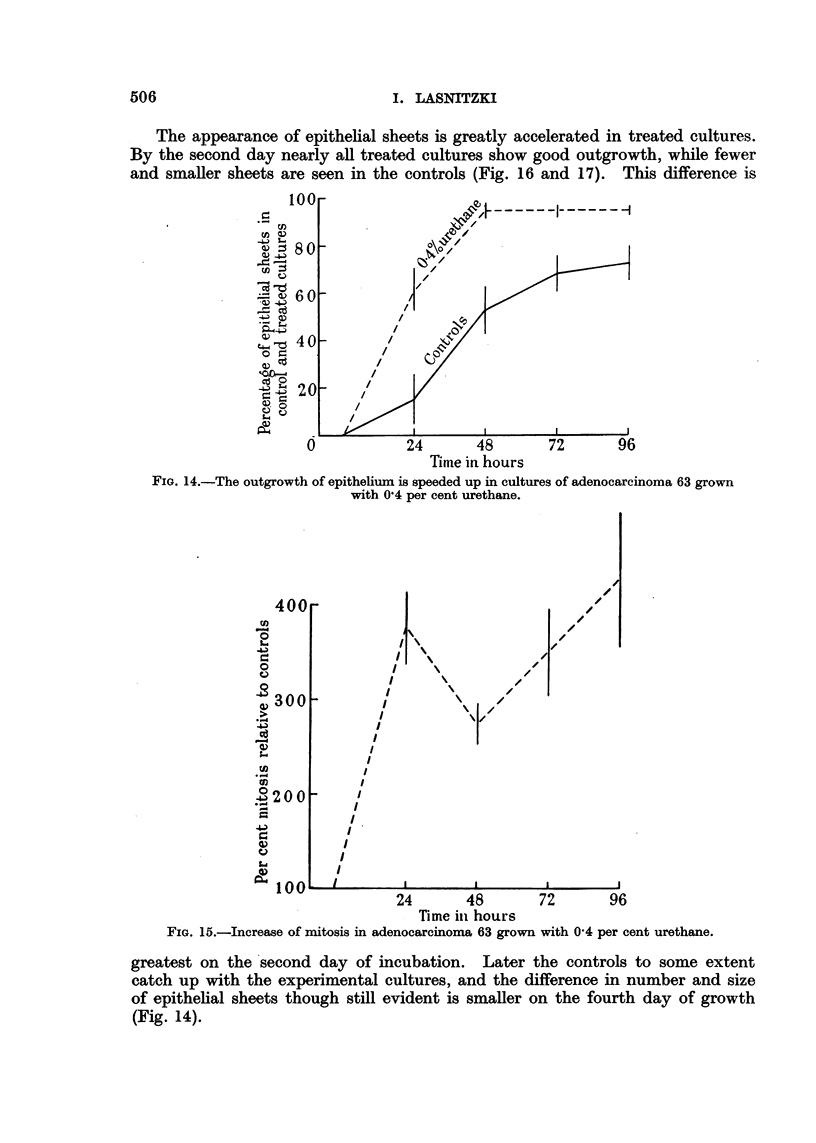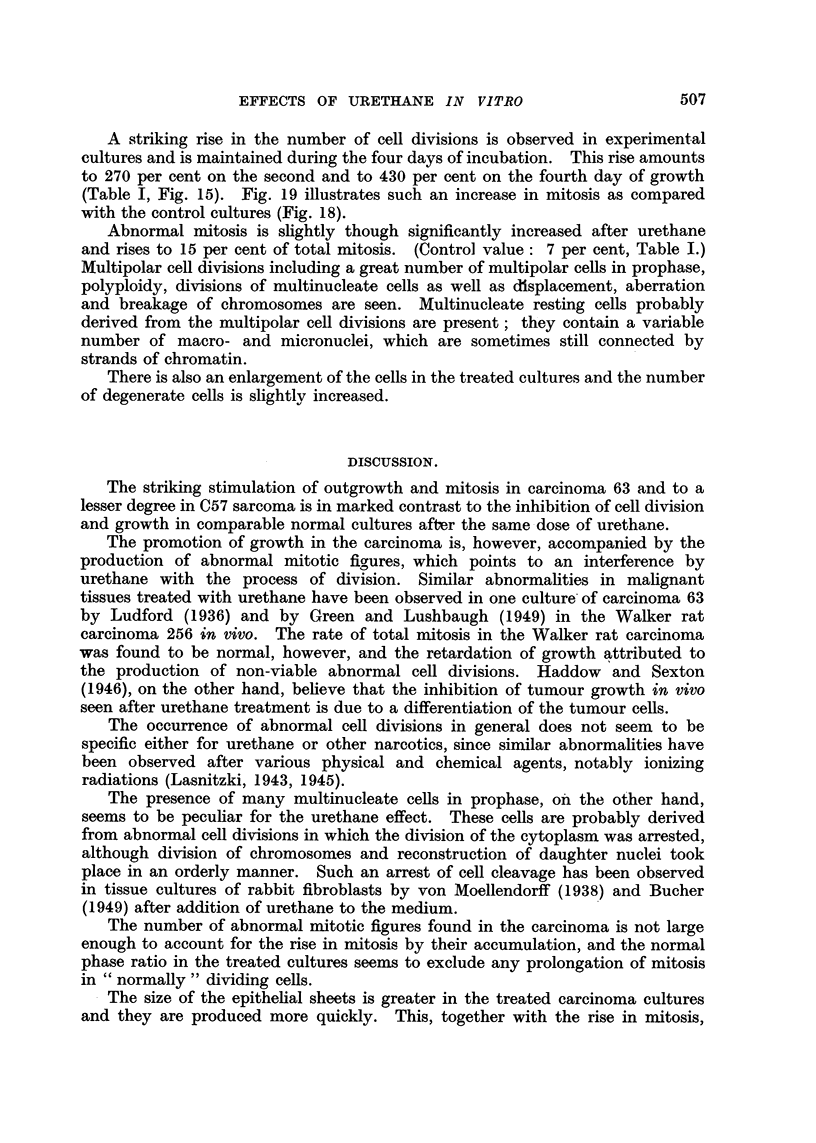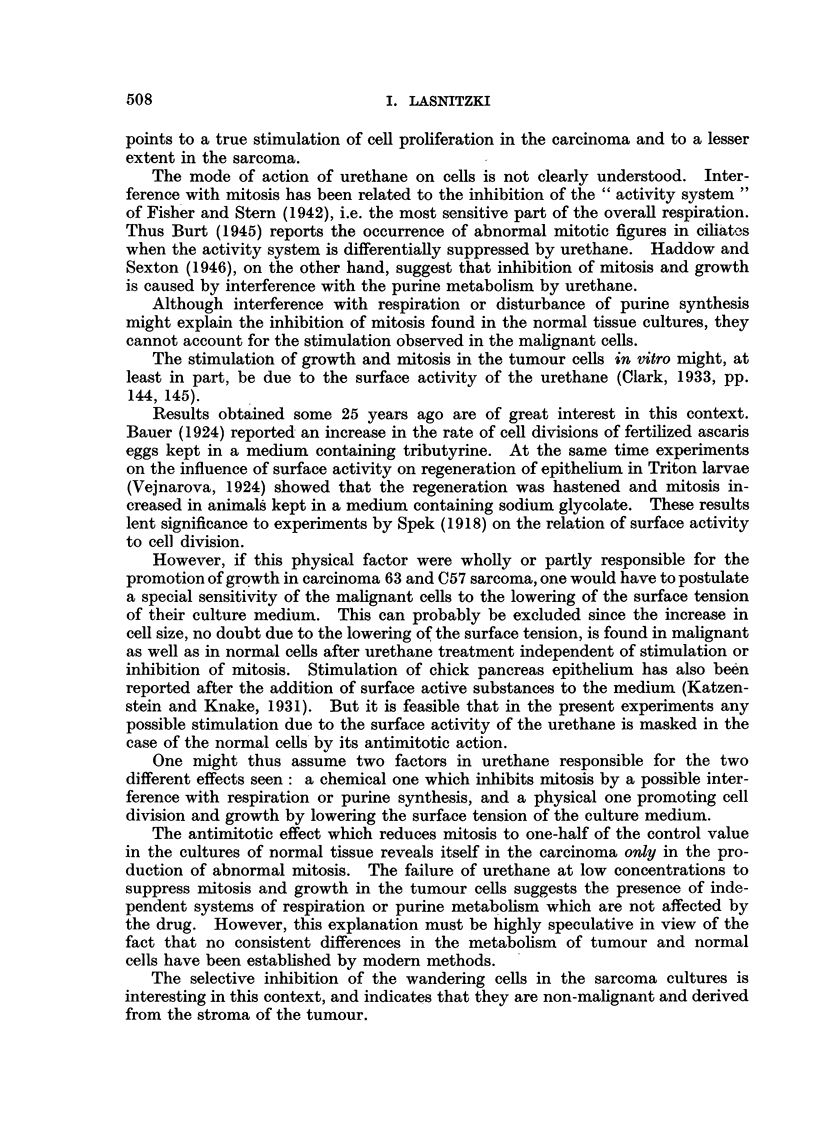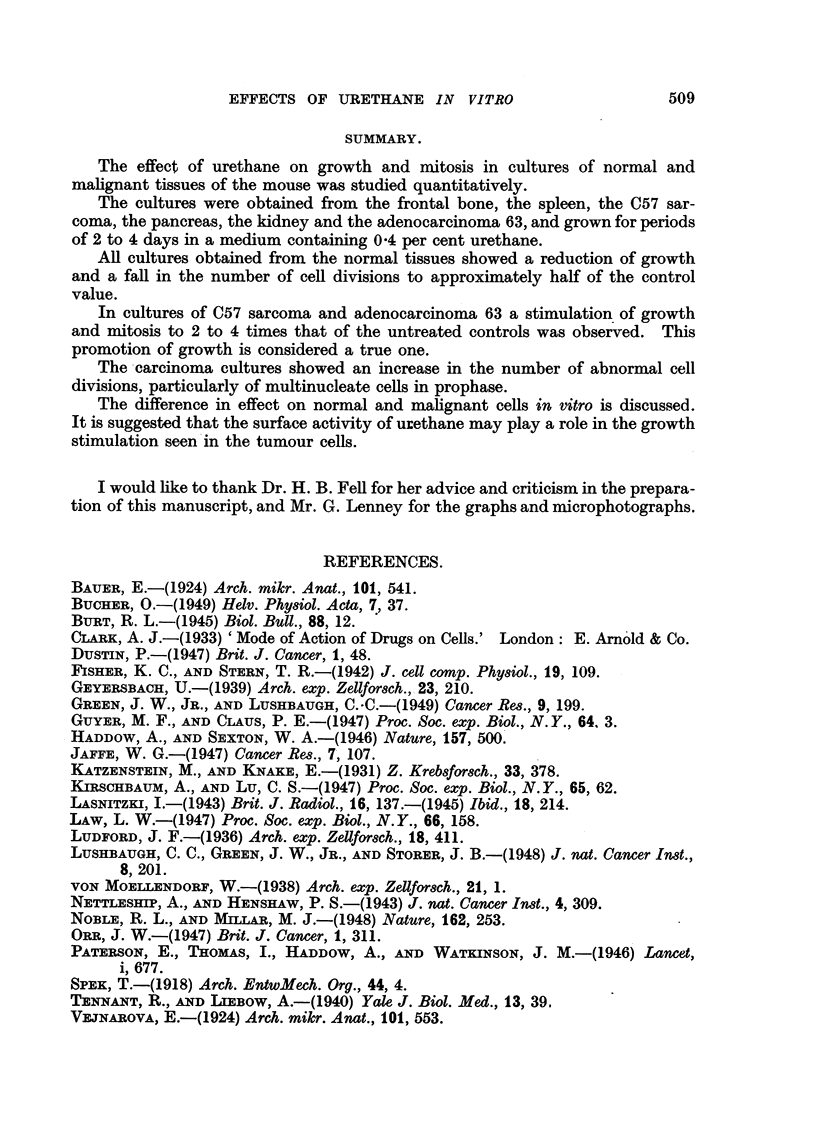# Some Effects of Urethane on the Growth and Mitosis of Normal and Malignant Cells In Vitro

**DOI:** 10.1038/bjc.1949.53

**Published:** 1949-12

**Authors:** I. Lasnitzki

## Abstract

**Images:**


REFERENCES.

### SOME EFFECTS OF URETHANE ON THE GROWTH AND MITOSIS OF NORMAL AND MALIGNANT CELLS *IN VITRO*.

I. LASNITZKI.*

*From the Strangeways Research Laboratory, Cambridge.*

Received for publication October 8, 1949.

DURING recent years the effect of urethane (ethylcarbamate) on various biological materials has been widely studied. Mitotic inhibition in tissue culture (Geyersbach, 1939; Bucher, 1949; Tennant and Liebow, 1940/41) and rat cornea (Guyer and Claus, 1947), has been observed as well as mitotic degenerations in the Lieberkuehn crypts of the intestine of the mouse, though not in thymus or lymph nodes (Dustin, 1947). Wound healing in rats (Lushbaugh, Green and Storer, 1948) and the growth *in vivo* of the Walker rat carcinoma 256

* Sir Halley Stewart Fellow.

has been retarded (Haddow and Sexton, 1946; Green and Lushbaugh, 1949). Ludford (1936) obtained rather inconsistent results with cultures of carcinoma 63 grown in a medium containing urethane, but found in one culture abnormal metaphases, an effect similar to that observed with colchicine.

The specific effect of urethane in mouse and human leukaemia is well known (Kirschbaum and Lu, 1947; Law, 1947; Paterson, Thomas, Haddow and Watkinson, 1946), but whether the reduction of the white blood cells is due to maturation or mitotic inhibition has not yet been decided.

The appearance of lung adenomata in mice and rats following urethane treatment (Nettleship and Henshaw, 1943; Jaffe, 1947; Orr, 1947) and of highly malignant lymphosarcomata and haemangiomata in mice (Noble and Millar, 1948) has been described. The question whether the production of tumours is due to any direct carcinogenic action of the drug on the bronchial epithelium or whether it is secondary to an unspecific irritation and inflammation is still open.

In view of the often inconsistent though apparently selective effects of the drug, it seemed desirable to study its action on growth and mitosis in various normal and malignant tissues of the mouse *in vitro* in order to find whether it affects specifically any given type of cell, and also to compare its action on normal and malignant cells.

The tissues chosen for the experiments were grouped according to the type of cells they produced, fibroblastic or epithelial. Cells of the fibroblast type derived from frontal bone, spleen, and a sarcoma were used in the first series, and epithelial cells from the pancreas, kidney medulla and adeno-carcinoma 63 in the second series.

### MATERIAL AND METHODS.

The cultures were obtained from the spleen, frontal bone, kidney medulla and pancreas of newly born mice, from the C57 sarcoma and the adenocarcinoma 63.

They were grown by the hanging drop technique in a mixture of rat and fowl plasma and chick embryo extract in equal parts. Urethane was added in Ringer solution to the medium of the experimental cultures, the final concentration in the medium being 0·4 per cent; a corresponding amount of Ringer solution was added to the medium of the control cultures. The cultures were then incubated and grown for periods of 2 to 4 days. Preliminary experiments showed the peak of mitosis to occur in the normal untreated cultures on the second day of incubation and usually cultures were fixed at that time. In addition, cultures of pancreas and carcinoma 63 were fixed on the fourth day of incubation. They were fixed in Susa's solution and stained with Mayer's or Ehrlich's haematoxylin and mounted whole.

Mitosis was assessed quantitatively by counting all the mitotic cells present in the zone of growth, and the result was expressed as a percentage inhibition or increase of the control value. Abnormal cell divisions, if present, were also counted and expressed as a percentage of total mitosis. The percentage of abnormal mitotic cells found in the controls was subtracted from that seen in the treated cultures. The counts shown in Table I thus refer to the increase seen in experimental cultures.

Each point represents the average of at least a dozen experimental and control cultures in the case of spleen, bone, C57 sarcoma and kidney medulla. For the experiments dealing with pancreas and carcinoma 63 approximately 50 cultures each have been used.

### RESULTS.

*Effect of urethane on normal fibroblastic growth* in vitro.

A. *Frontal bone.*—Fig. 1 shows a typical untreated culture from the frontal bone of the mouse on the second day of growth. By this time a dense zone of growth consisting of several layers of spindle-shaped cells surrounds the central explant. Mitosis is plentiful (Fig. 3). In cultures treated with urethane the size of the zone of growth is much smaller and less dense while mitosis is reduced to about half of the normal value (Fig. 2 and 4, Table I). The incidence of abnormal mitotic figures is not increased in urethane-treated cultures. The size of the resting cells shows a distinct enlargement in contrast to those seen in untreated cultures.

TABLE I.—*The Effect of* 0·4 *per cent of Urethane on Mitosis in Tissue Cultures of Normal and Malignant Cells of the Mouse.*

| Cultures obtained from— | Type of cells. | Mitosis as percentage of the control count. Period of growth. 48 hours. | 96 hours. | Increase of abnormal mitosis as percentage of total mitosis. |
|---|---|---|---|---|
| Frontal bone | Fibroblastic | 46 ± 8·5 | .. | 1·0 ± 0·7 |
| Spleen | ,, | 56 ± 4·2 | .. | 5·2 ± 3·3 |
| C57 Sarcoma | ,, | 172 ± 13·4 | .. | 3·0 ± 3·2 |
| Kidney medulla | Epithelial | 41 ± 10·0 | .. | .. |
| Pancreas | ,, | 38 ± 5·3 | 57 ± 4·3 | .. |
| Adenocarcinoma 63 | ,, | 273 ± 22·0 | 430 ± 72·5 | 8·0 ± 2·3 |

B. *Spleen.*—These cultures gave a similar result. The zone of growth is reduced and mitosis shows a decrease to 56 per cent of the controls, while abnormal cell divisions are absent. There is also an increase in cell size. A small number of degenerate cells can be observed in bone and spleen cultures after urethane treatment.

*Effect of urethane on C57 sarcoma.*

This was originally induced by methylcholanthrene and has been grafted through several generations of pure line C57 mice. It consists of spindle cells with a fair amount of stroma. The tumour grows vigorously *in vitro* and liquefies the culture medium strongly. After the first day of incubation a dense zone of growth consisting of spindle-shaped cells can be seen. Besides these spindle cells small round mobile cells wander out from the central explant towards the periphery of the culture ; on the first day of growth they form a halo around the zone of spindle cells and also lie in scattered groups above them (Fig. 5). These wandering cells degenerate quickly and disappear after the second or third day of growth.

Mitosis is plentiful on the first and second day of growth, but decreases from the third day onwards (Fig. 7).

In contrast to cultures of frontal bone and spleen sarcoma cultures treated with urethane show an increase in mitosis of 172 per cent as compared with the untreated controls ; there is no rise in the number of abnormal mitotic figures. The density of the zone of growth appears to be greater after the addition of urethane, although its area is not markedly enlarged. On the other hand, the wandering cells are absent (Fig. 6 and 8). The cell size is slightly increased after treatment with urethane.

An interesting feature is the absence of liquefaction in the experimental cultures which, in consequence, had a longer life span than their controls, which had to be changed every two days. As a rule, cultures derived from mammalian tissue need a transfer every 3 to 5 days.

There was no evidence of cell degeneration due to the urethane.

*Effect of urethane on normal epithelial growth.*

A. *Kidney medulla.*—In normal cultures on the second day of growth the explant develops a sheet of epithelium of varying density which shows a thickening of its edge. The cells are here packed more tightly and stain more deeply than the rest of the epithelium. Mitotic cells are scattered in the zone of growth, their rate amounting to approximately 3 to 4 per cent of resting cells.

In cultures treated with urethane the size of the sheet is greatly diminished and the peripheral bulge is missing. Mitosis is reduced to 41 per cent of the control value. There is no evidence of abnormal cell divisions, but a small number of degenerate cells can be observed. The cell size is also enlarged as compared with the untreated cultures.

B. *Pancreas.*—On the second day of growth about two-thirds of all cultures obtained from the mouse pancreas form sheets of columnar epithelium arranged

---

EXPLANATION OF PLATES.

Frontal bone after two days' growth.

FIG. 1 AND 3.—Peripheral part of control cultures. Note abundance of mitosis in Fig. 3. × 40 and × 350.

FIG. 2 AND 4.—Peripheral parts of similar cultures grown in a medium containing 0·4 per cent urethane. Note reduction of outgrowth and mitosis and increase in cell size. × 40 and × 350.

C57 sarcoma after two days' growth.

FIG. 5 AND 7.—Edge of control cultures showing groups of macrophages and scarcity of mitosis. × 100 and × 385.

FIG. 6 AND 8.—Edge of similar cultures grown with 0·4 per cent urethane. Note mitotic cells and absence of macrophages. × 100 and × 385.

Pancreas epithelium after two days' growth.

FIG. 9 AND 11.—Edge of control cultures. Note mitotic cells in Fig. 11. × 140 and × 350.

FIG. 10 AND 12.—Edge of similar cultures grown with 0·4 per cent urethane. Note scarcity of mitosis and presence of degenerate cells. × 140 and × 350.

Adenocarcinoma 63 after one day's growth.

FIG. 16.—Control culture. × 60.

FIG. 17.—Culture grown with 0·4 per cent urethane. × 60.

FIG. 18.—Edge of control culture. × 350.

FIG. 19.—Periphery of a similar culture grown with 0·4 per cent urethane. Note abundance of mitosis. × 350.

Lasnitzki.

9

10

11

12

16 17

18 19

Lasnitzki.

in a single layer (Fig. 9). A few fibroblasts are, as a rule, present in these cultures. In the remaining third of the cultures fibroblastic growth only is obtained.

Mitotic cells are scattered throughout the outgrowth, and amount to about 4 per cent of the resting cell population (Fig. 11).

The addition of urethane has no effect on the number of cultures forming epithelial sheets. The size of the sheets, however, is much reduced in the treated cultures and only small epithelial islets are produced, while in the controls the explants are entirely surrounded by epithelium (Fig. 10 and 12). At the same time mitosis is reduced to 38 per cent of normal on the second day and to 57 per cent of normal on the fourth day of growth (Table I, Fig. 13). Abnormal mitotic figures were absent, but a small number of pycnotic cells could be seen in the treated cultures.

FIG. 13.—Percentage inhibition of mitosis in cultures of mouse pancreas with 0·4 per cent urethane.

*Effect of urethane on adenocarcinoma* 63.

This adenocarcinoma is a transplantable, rapidly growing, small-celled tumour showing little stroma, and grows readly *in vitro*. After two days' incubation the explant is surrounded by a zone of growth consisting of a sheet of polygonal cells. This sheet is several layers thick near the explant, but it thins out towards the periphery to form a single layer. Mitotic figures are scattered throughout the zone of growth but are more frequent in the neighbourhood of the explant than in the periphery of the outgrowth. Between the first and third day of incubation about 5 per cent of the total cell population are in division at any given moment. Abnormal mitotic figures are seen but as a rule they do not exceed 7 per cent of total mitosis. Some degenerate cells are always present in normal cultures (Fig. 18).

The response to the addition of urethane was a marked stimulation of outgrowth and mitosis.

The appearance of epithelial sheets is greatly accelerated in treated cultures. By the second day nearly all treated cultures show good outgrowth, while fewer and smaller sheets are seen in the controls (Fig. 16 and 17). This difference is

FIG. 14.—The outgrowth of epithelium is speeded up in cultures of adenocarcinoma 63 grown with 0·4 per cent urethane.

FIG. 15.—Increase of mitosis in adenocarcinoma 63 grown with 0·4 per cent urethane.

greatest on the second day of incubation. Later the controls to some extent catch up with the experimental cultures, and the difference in number and size of epithelial sheets though still evident is smaller on the fourth day of growth (Fig. 14).

A striking rise in the number of cell divisions is observed in experimental cultures and is maintained during the four days of incubation. This rise amounts to 270 per cent on the second and to 430 per cent on the fourth day of growth (Table I, Fig. 15). Fig. 19 illustrates such an increase in mitosis as compared with the control cultures (Fig. 18).

Abnormal mitosis is slightly though significantly increased after urethane and rises to 15 per cent of total mitosis. (Control value : 7 per cent, Table I.) Multipolar cell divisions including a great number of multipolar cells in prophase, polyploidy, divisions of multinucleate cells as well as displacement, aberration and breakage of chromosomes are seen. Multinucleate resting cells probably derived from the multipolar cell divisions are present ; they contain a variable number of macro- and micronuclei, which are sometimes still connected by strands of chromatin.

There is also an enlargement of the cells in the treated cultures and the number of degenerate cells is slightly increased.

### DISCUSSION.

The striking stimulation of outgrowth and mitosis in carcinoma 63 and to a lesser degree in C57 sarcoma is in marked contrast to the inhibition of cell division and growth in comparable normal cultures after the same dose of urethane.

The promotion of growth in the carcinoma is, however, accompanied by the production of abnormal mitotic figures, which points to an interference by urethane with the process of division. Similar abnormalities in malignant tissues treated with urethane have been observed in one culture of carcinoma 63 by Ludford (1936) and by Green and Lushbaugh (1949) in the Walker rat carcinoma 256 *in vivo.* The rate of total mitosis in the Walker rat carcinoma was found to be normal, however, and the retardation of growth attributed to the production of non-viable abnormal cell divisions. Haddow and Sexton (1946), on the other hand, believe that the inhibition of tumour growth *in vivo* seen after urethane treatment is due to a differentiation of the tumour cells.

The occurrence of abnormal cell divisions in general does not seem to be specific either for urethane or other narcotics, since similar abnormalities have been observed after various physical and chemical agents, notably ionizing radiations (Lasnitzki, 1943, 1945).

The presence of many multinucleate cells in prophase, on the other hand, seems to be peculiar for the urethane effect. These cells are probably derived from abnormal cell divisions in which the division of the cytoplasm was arrested, although division of chromosomes and reconstruction of daughter nuclei took place in an orderly manner. Such an arrest of cell cleavage has been observed in tissue cultures of rabbit fibroblasts by von Moellendorff (1938) and Bucher (1949) after addition of urethane to the medium.

The number of abnormal mitotic figures found in the carcinoma is not large enough to account for the rise in mitosis by their accumulation, and the normal phase ratio in the treated cultures seems to exclude any prolongation of mitosis in " normally " dividing cells.

The size of the epithelial sheets is greater in the treated carcinoma cultures and they are produced more quickly. This, together with the rise in mitosis, points to a true stimulation of cell proliferation in the carcinoma and to a lesser extent in the sarcoma.

The mode of action of urethane on cells is not clearly understood. Interference with mitosis has been related to the inhibition of the " activity system " of Fisher and Stern (1942), i.e. the most sensitive part of the overall respiration. Thus Burt (1945) reports the occurrence of abnormal mitotic figures in ciliates when the activity system is differentially suppressed by urethane. Haddow and Sexton (1946), on the other hand, suggest that inhibition of mitosis and growth is caused by interference with the purine metabolism by urethane.

Although interference with respiration or disturbance of purine synthesis might explain the inhibition of mitosis found in the normal tissue cultures, they cannot account for the stimulation observed in the malignant cells.

The stimulation of growth and mitosis in the tumour cells *in vitro* might, at least in part, be due to the surface activity of the urethane (Clark, 1933, pp. 144, 145).

Results obtained some 25 years ago are of great interest in this context. Bauer (1924) reported an increase in the rate of cell divisions of fertilized ascaris eggs kept in a medium containing tributyrine. At the same time experiments on the influence of surface activity on regeneration of epithelium in Triton larvae (Vejnarova, 1924) showed that the regeneration was hastened and mitosis increased in animals kept in a medium containing sodium glycolate. These results lent significance to experiments by Spek (1918) on the relation of surface activity to cell division.

However, if this physical factor were wholly or partly responsible for the promotion of growth in carcinoma 63 and C57 sarcoma, one would have to postulate a special sensitivity of the malignant cells to the lowering of the surface tension of their culture medium. This can probably be excluded since the increase in cell size, no doubt due to the lowering of the surface tension, is found in malignant as well as in normal cells after urethane treatment independent of stimulation or inhibition of mitosis. Stimulation of chick pancreas epithelium has also been reported after the addition of surface active substances to the medium (Katzenstein and Knake, 1931). But it is feasible that in the present experiments any possible stimulation due to the surface activity of the urethane is masked in the case of the normal cells by its antimitotic action.

One might thus assume two factors in urethane responsible for the two different effects seen : a chemical one which inhibits mitosis by a possible interference with respiration or purine synthesis, and a physical one promoting cell division and growth by lowering the surface tension of the culture medium.

The antimitotic effect which reduces mitosis to one-half of the control value in the cultures of normal tissue reveals itself in the carcinoma *only* in the production of abnormal mitosis. The failure of urethane at low concentrations to suppress mitosis and growth in the tumour cells suggests the presence of independent systems of respiration or purine metabolism which are not affected by the drug. However, this explanation must be highly speculative in view of the fact that no consistent differences in the metabolism of tumour and normal cells have been established by modern methods.

The selective inhibition of the wandering cells in the sarcoma cultures is interesting in this context, and indicates that they are non-malignant and derived from the stroma of the tumour.

SUMMARY.

The effect of urethane on growth and mitosis in cultures of normal and malignant tissues of the mouse was studied quantitatively.

The cultures were obtained from the frontal bone, the spleen, the C57 sarcoma, the pancreas, the kidney and the adenocarcinoma 63, and grown for periods of 2 to 4 days in a medium containing 0·4 per cent urethane.

All cultures obtained from the normal tissues showed a reduction of growth and a fall in the number of cell divisions to approximately half of the control value.

In cultures of C57 sarcoma and adenocarcinoma 63 a stimulation of growth and mitosis to 2 to 4 times that of the untreated controls was observed. This promotion of growth is considered a true one.

The carcinoma cultures showed an increase in the number of abnormal cell divisions, particularly of multinucleate cells in prophase.

The difference in effect on normal and malignant cells *in vitro* is discussed. It is suggested that the surface activity of urethane may play a role in the growth stimulation seen in the tumour cells.

I would like to thank Dr. H. B. Fell for her advice and criticism in the preparation of this manuscript, and Mr. G. Lenney for the graphs and microphotographs.

REFERENCES.

Bauer, E.—(1924) *Arch. mikr. Anat.*, **101**, 541.
Bucher, O.—(1949) *Helv. Physiol. Acta*, **7**, 37.
Burt, R. L.—(1945) *Biol. Bull.*, **88**, 12.
Clark, A. J.—(1933) 'Mode of Action of Drugs on Cells.' London : E. Arnold & Co.
Dustin, P.—(1947) *Brit. J. Cancer*, **1**, 48.
Fisher, K. C., and Stern, T. R.—(1942) *J. cell comp. Physiol.*, **19**, 109.
Geyersbach, U.—(1939) *Arch. exp. Zellforsch.*, **23**, 210.
Green, J. W., Jr., and Lushbaugh, C. C.—(1949) *Cancer Res.*, **9**, 199.
Guyer, M. F., and Claus, P. E.—(1947) *Proc. Soc. exp. Biol., N.Y.*, **64**, 3.
Haddow, A., and Sexton, W. A.—(1946) *Nature*, **157**, 500.
Jaffe, W. G.—(1947) *Cancer Res.*, **7**, 107.
Katzenstein, M., and Knake, E.—(1931) *Z. Krebsforsch.*, **33**, 378.
Kirschbaum, A., and Lu, C. S.—(1947) *Proc. Soc. exp. Biol., N.Y.*, **65**, 62.
Lasnitzki, I.—(1943) *Brit. J. Radiol.*, **16**, 137.—(1945) *Ibid.*, **18**, 214.
Law, L. W.—(1947) *Proc. Soc. exp. Biol., N.Y.*, **66**, 158.
Ludford, J. F.—(1936) *Arch. exp. Zellforsch.*, **18**, 411.
Lushbaugh, C. C., Green, J. W., Jr., and Storer, J. B.—(1948) *J. nat. Cancer Inst.*, **8**, 201.
von Moellendorf, W.—(1938) *Arch. exp. Zellforsch.*, **21**, 1.
Nettleship, A., and Henshaw, P. S.—(1943) *J. nat. Cancer Inst.*, **4**, 309.
Noble, R. L., and Millar, M. J.—(1948) *Nature*, **162**, 253.
Orr, J. W.—(1947) *Brit. J. Cancer*, **1**, 311.
Paterson, E., Thomas, I., Haddow, A., and Watkinson, J. M.—(1946) *Lancet*, i, 677.
Spek, T.—(1918) *Arch. EntwMech. Org.*, **44**, 4.
Tennant, R., and Liebow, A.—(1940) *Yale J. Biol. Med.*, **13**, 39.
Vejnarova, E.—(1924) *Arch. mikr. Anat.*, **101**, 553.